# National Distribution of Bisexual and Parthenogenetic *Haemaphysalis longicornis* of Japan, and a Real‐Time PCR–Based Method to Distinguish the Two Reproductive Groups

**DOI:** 10.1155/japr/9395344

**Published:** 2026-07-31

**Authors:** Mizue Inumaru, Rio Noda, Kentaro Itokawa, Ryo Matsumura, Toshiya Kimura, Ryusei Kuwata, Satoko Nakao, Osamu Tsuha, Akihiro Takemura, Shoko Nishiyama, Takeo Yamauchi, Hiroo Tamatani, Akira Yoshikawa, Mitsuru Hattori, Toshinori Sasaki, Mamoru Watanabe, Kyoko Sawabe, Alisa Rose Aboshi, Daisuke Kobayashi, Chao Yang, Aiki Yamada, Haruhiko Isawa, Shinji Kasai, Yukiko Higa

**Affiliations:** ^1^ Department of Medical Entomology, National Institute of Infectious Diseases, Japan Institute for Health Security, Shinjuku, Tokyo, Japan, nih.go.jp; ^2^ School of Agriculture, Meiji University, Kawasaki, Kanagawa, Japan, meiji.ac.jp; ^3^ Meat Inspection Center of Ehime, Ehime Prefectural Government, Ōzu, Ehime, Japan; ^4^ Faculty of Veterinary Medicine, Okayama University of Science, Imabari, Ehime, Japan, ous.ac.jp; ^5^ Yaeyama Livestock Hygiene Service Center of Okinawa Prefecture, Ishigaki, Okinawa, Japan; ^6^ National Research Center for Protozoan Diseases, Obihiro University of Agriculture and Veterinary Medicine, Obihiro, Hokkaido, Japan, obihiro.ac.jp; ^7^ Division of Virology and Medical Zoology, Chiba Prefectural Institute of Public Health, Chiba, Chiba, Japan; ^8^ Department of Virology I, National Institute of Infectious Diseases, Japan Institute for Health Security, Shinjuku, Tokyo, Japan, nih.go.jp; ^9^ Laboratory of Entomology, Obihiro University of Agriculture and Veterinary Medicine, Obihiro, Hokkaido, Japan, obihiro.ac.jp; ^10^ Department of Bear Management, Picchio Wildlife Research Center, Karuizawa, Nagano, Japan; ^11^ Public Health and Hygiene Research Department, Nagasaki Prefectural Institute of Environmental and Public Health, Omura, Nagasaki, Japan; ^12^ Graduate School of Integrated Science and Technology, Nagasaki University, Nagasaki, Nagasaki, Japan, nagasaki-u.ac.jp; ^13^ Laboratory of Molecular Immunology, Department of Animal Resource Sciences, Graduate School of Agricultural and Life Sciences, The University of Tokyo, Bunkyo, Tokyo, Japan, u-tokyo.ac.jp

**Keywords:** *Haemaphysalis longicornis*, identification method, Ixodidae, Japan, national distribution, parthenogenesis, real-time PCR

## Abstract

The Asian longhorned tick, *Haemaphysalis longicornis*, is a well‐known vector of several zoonoses including SFTS and Japanese spotted fever. This species consists of two different reproductive forms, bisexual and parthenogenetic, which are suggested to have differential roles in disease transmission. In Japan, the distributions of these forms have been suspected from sex bias in each population. Meanwhile, recent insights about the origin of the parthenogenetic form suggest the possibility of individual‐level diagnosis using mitochondrial phylogeny. To reassess the current distribution of those reproductive forms and potential changes over time in Japan, 1750 *H. longicornis* collected across 23 prefectures of Japan were investigated. The *COI* barcoding region was used to distinguish the mitochondrial haplogroup of each individual. Additionally, we developed real‐time PCR probes to distinguish the two mitochondrial haplogroups representing each reproductive form. As previous studies have suggested, the parthenogenetic mitochondrial haplogroup was present throughout the country, while the bisexual mitochondrial haplogroup was found only in central and western Japan. Interestingly, populations consisting of either one or both haplogroups were found in neighboring areas, highlighting the need for more detailed and fine‐scaled investigations in order to fully reveal the distribution of the reproductive groups. The newly developed real‐time PCR method successfully differentiated all tested haplotypes. Furthermore, alignment of 173 *COI* haplotypes revealed that the diagnostic nucleotide selected for the method was consistent across all haplotypes, including those outside of Japan. This suggests that this method can accurately distinguish between the two haplogroups of *H. longicornis* in both Japan and other countries. This one‐step method will serve as a rapid tool to distinguish bisexual and parthenogenetic *H. longicornis*, making further investigations, particularly into their role in disease transmission and shifts in distributions, possible.

## 1. Introduction

The Asian longhorned tick, *Haemaphysalis longicornis*, is known as an important vector species for several zoonotic tick‐borne diseases including severe fever with thrombocytopenia syndrome (SFTS) and Japanese spotted fever [1–3]. The species has rapidly spread its distribution within the last few decades, expanding from its native range in eastern Asia to distant areas such as Australia, New Zealand, North America, and Turkey [2, 4, 5]. One of the major factors that has contributed to this rapid expansion is likely the presence of parthenogenetic populations, which are triploid and capable of reproducing without males [6, 7]. In fact, newly established areas outside of eastern Asia consist solely of parthenogenetic populations [3, 4]. On the other hand, both bisexual and parthenogenetic populations are found in the native areas of eastern Asia [1, 3, 6].

A previous study suggested that the two reproductive forms have differential roles in disease transmission [1], highlighting the need to monitor the distribution of each reproductive group. In Japan, where *H. longicornis* is distributed throughout the country, the presence and absence of males had been classically used to speculate on the distributions of the parthenogenetic form [8]. However, this approach does not consider the sympatry of both groups, which has been described in China and Japan [1, 9]. Consequently, reproductive groups could only be speculated at the population level, and the “true” distributions of the two groups had not been accurately understood. Furthermore, several factors such as the range expansion of host mammals, climate change, and changes in human activities are known to impact tick densities and distributions [3, 10–12]. Hence, a re‐evaluation of the distribution of bisexual and parthenogenetic populations of *H. longicornis* at the individual level is necessary. Recent studies have shown that reproductive groups can be distinguished by flow cytometry [1, 13], using differences in ploidy and single‐nucleotide polymorphism (SNP) genotypes [9]. Furthermore, the whole or *COI* barcoding region of the mitochondrial genome has been suggested as a useful molecular marker based on the assumption that the parthenogenetic populations have a single evolutionary origin and monophyletic mitochondrial lineage [1, 9].

A recent study investigated the distribution and genetic population structure of *H. longicornis* in Japan [14]. Meanwhile, several areas have still been uninvestigated. In this study, we investigated the *COI* barcoding region of *H. longicornis* in Japan to reassess the current distribution of the two reproductive forms and potential changes over time. Additionally, a real‐time PCR–based method was developed based on a fixed SNP in the *COI* barcoding region to distinguish the two reproductive groups of *H. longicornis*.

## 2. Materials and Methods

### 2.1. Tick Collection and Confirmation of Reproductive Group

**Figure 1:**
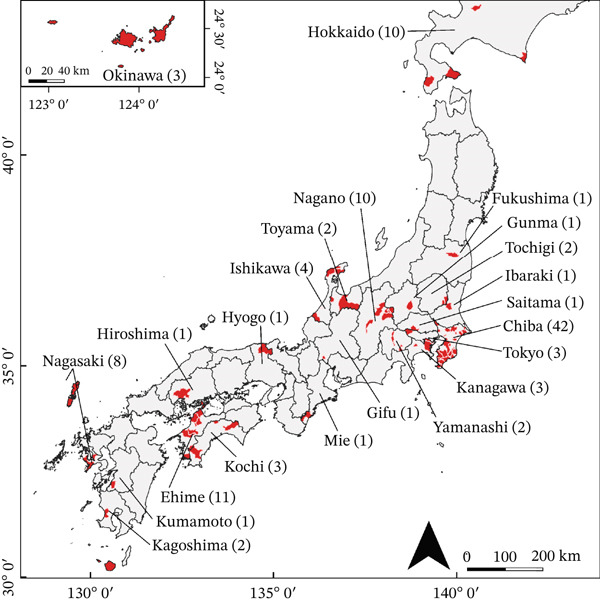
Map of the sampling locations of *Haemaphysalis longicornis*. The prefectures where samples were obtained are labeled. The number of sampling sites is shown in parentheses. The cities or towns where samples were obtained are shown in red.

Ticks were collected throughout Japan from 2013 to 2025 for multiple independent studies and surveillance purposes. Most ticks were collected by the flagging method, while some were collected directly from host animals. After morphological identification using a stereo microscope (Olympus‐SZ61, Japan) and a light microscope (Nikon Eclipse Ci, Japan), ticks identified as *H. longicornis* were used for subsequent analyses. In total, 1750 *H. longicornis* were collected from 114 sites of 72 localities in 23 prefectures between 2013 and 2025 (Figure 1; Table S1). DNA was extracted, and *COI* haplotypes were confirmed as previously described [9]. Briefly, template DNA was prepared by clean extraction using the Quick‐DNA/RNA MagBead Kit (Zymo Research) or crude extraction using 0.2 M NaOH, depending on sample availability. For samples in which the tick bodies are to be pooled for virus detection or other analyses, legs were individually removed and processed using NaOH. All whole‐body samples were processed with the kit. The partial *COI* gene of ticks was confirmed by PCR, using the primers dgLCO1490 and dgHCO2198 [15]. PCR products were purified with Agencourt AMPure XP beads (Beckman Coulter, Tokyo, Japan) and sequenced by Azenta (Tokyo, Japan). The obtained *COI* haplotypes were trimmed with the ATGC software (Nihon Server Corp., Tokyo, Japan) and multiple aligned with sequences from a previous study [1] using GENETYX Ver. 13 (Nihon Server Corp.). A phylogenetic tree was constructed using the maximum‐likelihood method and the Tamura‐3‐parameter model with Gamma distributed with invariant sites and 1000 bootstrap replicates, selected in MEGA X [16] as the best‐fit model. Results from the phylogenetic analysis were used to distinguish the reproductive haplogroup of the obtained haplotypes.

### 2.2. Distribution Map

A distribution map was generated using QGIS 3.30.0 software and administrative boundary data obtained from the National Land Numerical Information of Japan′s Ministry of Land, Infrastructure, Transport and Tourism (MLIT). Data from this study and from previous studies were incorporated [9, 14]. Furthermore, distribution maps from older studies [8, 17] were recreated using the georeferencer plugin.

### 2.3. Primer and Probe Design

*COI* haplotypes (accession nos. LC860032–LC860063) from a previous study [9] were used to design specific primers and probes. The haplotypes were aligned and trimmed using the ATGC software. Using the aligned haplotypes, candidate diagnostic nucleotide positions (i.e., positions that were consistently different among bisexual and parthenogenetic haplotypes) were selected. Among these candidate positions, one position identified at position 1270 of the *COI* gene (aligned to GenBank accession number MW642371.1; bisexual haplotypes with C, parthenogenetic haplotypes with T) with minimum variations in the surrounding bases was chosen as the target position.

Aligned haplotypes were further trimmed down to the first 160 bp encompassing the chosen candidate and used for Primer‐BLAST [18]. PCR product size was set to a minimum of 100 and a maximum of 150; other parameters were kept as default. A candidate primer set was chosen, and oligonucleotide primers were designed, including degenerate bases to include haplotype variations: Hlongi_coi_F6 (5 ^′^‐GAA CTA GGG CAA CCT GGY ACA‐3 ^′^) and Hlongi_coi_R6 (5 ^′^‐ACT AAT CAA TTT CCA AAT CCR CCA‐3 ^′^). Specific amplification was confirmed by qPCR, followed by a melt curve analysis using QuantStudio 1 (Thermo Fisher Scientific K.K., Tokyo, Japan). Twelve bisexual and 10 parthenogenetic *H. longicornis* from a previous study [9] were randomly selected. Each reaction was carried out in a final volume of 10 *μ*L containing the following: 0.4 *μ*M of deoxynucleotide triphosphate (dNTP), 1x KOD FX buffer (Toyobo, Japan), 0.2 U KOD FX (Toyobo), 0.25 *μ*M per primer, 1x EvaGreen (Biotium Inc., Hayward, California), and 1 *μ*L of template DNA. The cycling conditions were as follows: initial denaturing at 94°C for 2 min and then 40 cycles of 98°C for 10 s, 60°C for 30 s, and 68°C for 45 s. Following amplification, a melting curve analysis was performed by increasing the temperature from 60°C to 95°C at a ramp rate of 0.15°C/s, with continuous fluorescence monitoring.

Probe sequences were selected, analyzed, and optimized using the IDT OligoAnalyzer (Integrated DNA Technologies, Tokyo, Japan). Affinity Plus probes were synthesized by Integrated DNA Technologies (IDT). The following two probes were designed, including the diagnostic nucleotide position: Hlongi_coi_bi_probe1: 5 ^′^‐6‐FAM‐T GGA AAT G+A+C+CAA ATC T‐IBFQ‐3 ^′^ labeled with 5 ^′^ 6‐FAM and Hlongi_coi_par_probe1: 5 ^′^‐HEX‐T GGA AAT G+A+T+CAA ATC T‐IBFQ‐3 ^′^. Plus symbols (“+”) within the sequences indicate modified nucleotides for enhanced stability and binding efficiency.

### 2.4. Real‐Time PCR Assay

Real‐time PCR assays were performed using QuantStudio 1 (Thermo Fisher Scientific K.K.) in a total volume of 10 *μ*L containing 1x PrimeTime Gene Expression Master Mix (IDT), 0.5 *μ*M per primer, 0.5 *μ*M per probe, and 2 *μ*L of template DNA. The cycling conditions were as follows: initial denaturing at 95°C for 2 min and then 30 or 40 cycles of 95°C for 15 s, 58°C for 15 s, and 68°C for 45 s, during which data were collected using fluorescence detection. The ramp rate between temperature transitions was set to 1.6°C/s. Fluorescence signals from FAM and HEX dyes were detected in separate channels. Data was analyzed in the Design & Analysis software 2.6.0 (Thermo Fisher Scientific K.K.), and allelic discrimination plots were generated for genotyping.

### 2.5. Sensitivity and Specificity of the Real‐Time PCR Assay

To test the sensitivity of the real‐time PCR assay, *COI* haplotypes obtained from the *H. longicornis* collected in this study and a previous study [9] were tested using the protocol described above. One to nine individuals of each haplotype were randomly selected (176 individuals in total; Tables S1 and S2). Additionally, the validity of the selected diagnostic nucleotide position was checked across all haplotypes, including those from previous studies of Japan and other countries (Table S2). The haplotypes obtained in this study were multiple aligned with sequences from previous studies [1, 4, 9, 14, 19–27] using GENETYX Ver. 13, and the alignment was trimmed to include only shared regions. A phylogenetic tree was constructed in the same manner as described above, and nucleotides were compared between the bisexual and parthenogenetic haplogroups.

To confirm the specificity of the real‐time PCR assay, species other than *H. longicornis* were also tested. Four individuals each of nine *Haemaphysalis* species collected from five prefectures of Japan were used (Table S3). Morphological identification, DNA extraction, and real‐time PCR assay were carried out using the same methods as described above.

## 3. Results

### 3.1. Distribution of the Two Mitochondrial Haplogroups

**Figure 2:**
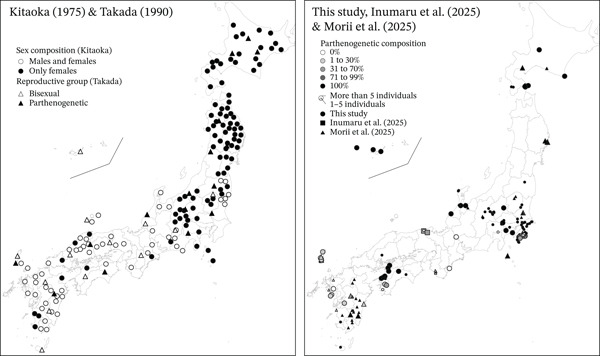
Distribution map of *H. longicornis* in Japan. (a) The map is recreated from figures in previous studies [8, 17]. (b) The map is created based on data from the present study and from previous studies utilizing *COI* barcoding for identification of the reproductive groups [9, 14]. Note that in the recreated maps, points do not show sample sizes.

Ninety‐five unique *COI* haplotypes were confirmed from the 1750 ticks collected throughout Japan, of which 65 and 30 were placed in the bisexual and parthenogenetic haplogroups by phylogenetic analysis, respectively (Figure S1). However, note that seven haplotypes were placed between the well‐defined clades containing parthenogenetic and bisexual haplotypes defined by Zhang et al. [1]. Consequently, these haplotypes were considered undefined and excluded from the distribution maps. All males were placed in the bisexual haplogroup, while nymphs and females were placed in either haplogroup (Table 1; Figure S1). Combining molecular data from previous studies [9, 14], individuals of the parthenogenetic haplogroup (hereinafter called parthenogenetic individuals) and individuals of the bisexual haplogroup (hereinafter called bisexual individuals) were found in 25 and 15 prefectures, respectively, out of the 28 prefectures investigated (Figure 2; Figures S2 and S3, Tables S1 and S2). Like previous studies have shown [8, 17], parthenogenetic individuals were found throughout the country, while bisexual individuals were only found from central to southern Japan. Meanwhile, although Takada [17] showed the presence of a bisexual population on Ishigaki Island of Okinawa Prefecture, only parthenogenetic individuals were found in this study. Additionally, several localities in Kagoshima Prefecture and Miyazaki Prefecture of the Kyushu area revealed completely parthenogenetic populations, while Takada [17] considered these prefectures to include mostly bisexual populations. In several locations, coexistence of both populations was confirmed. Notably, coexisting populations and populations consisting of only one reproductive haplogroup were found in proximity.

**Table 1:** Summary of *Haemaphysalis longicornis* ticks collected in this study and previous studies in Japan, by prefecture, stage, and reproductive group.

Prefecture	Parthenogenetic			Bisexual				Total			
	Nymph	Female	Male	Larva	Nymph	Female	Male	Larva	Nymph	Female	Male
Hokkaido	118	33							118	33	
Fukushima	5								5		
Ibaraki	1								1		
Tochigi	1	1							1	1	
Gunma	1	19							1	19	
Saitama	1								1		
Chiba	51	21		1	33	7	19	1	84	28	19
Tokyo	23								23		
Kanagawa	21	18							21	18	
Toyama	44	2							44	2	
Ishikawa	15	1					1		15	1	1
Yamanashi	4	1				1			4	2	
Nagano	9	4							9	4	
Gifu						2	14			2	14
Mie					6				6		
Hyogo							2				2
Hiroshima		1								1	
Ehime	267	105			10	6	16		277	111	16
Kochi	10	17			11	8	4		21	25	4
Nagasaki	39	5		9	588	40	49	9	627	45	49
Kumamoto		6			1	17	8		1	23	8
Kagoshima	1	1							1	1	
Okinawa	48	3							48	3	
Total	659	238	0	10	649	81	113	10	1,308	319	113

### 3.2. Evaluation of the Real‐Time PCR–Based Classification of Mitochondrial Lineages

**Figure 3:**
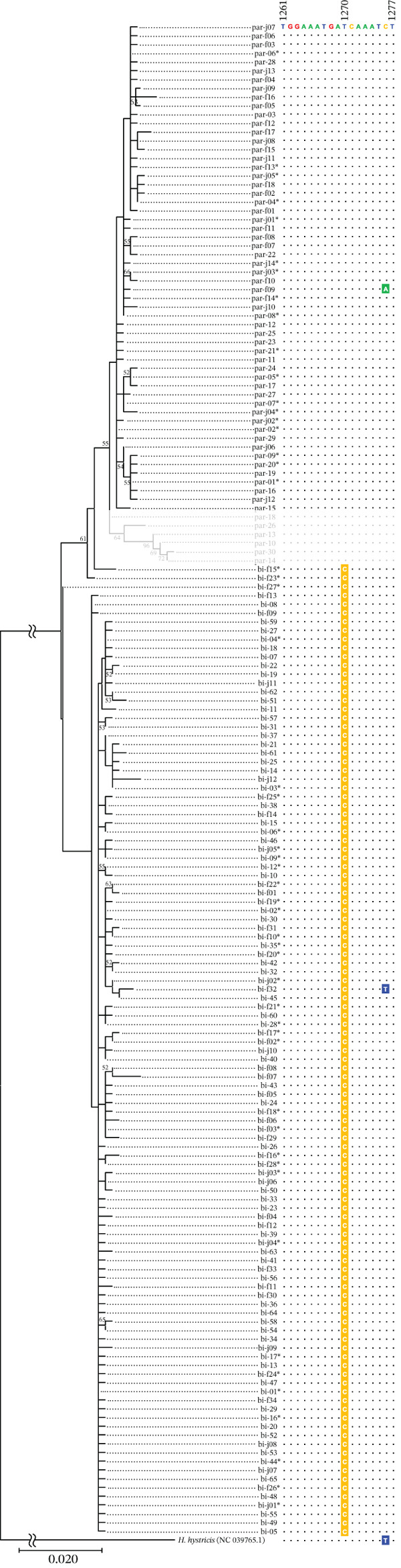
Maximum likelihood tree of *COI* sequences (602 bp) of *Haemaphysalis longicornis*, constructed using the Tamura‐3‐parameter model, and multiple alignment of the probe region of the real‐time PCR assay developed in this study. Bisexual and parthenogenetic groups are marked based on haplotypes from a previous study [1]. Haplotypes in which the ploidy has been confirmed by flow cytometry or SNP analysis are labeled with an asterisk (∗). Haplotypes that could not be defined to a specific haplogroup are shown in light color. *Haemaphysalis hystricis* was used as an outgroup. In the multiple alignment, dots (“・”) indicate positions identical to the top haplotype (par‐j07), while nucleotide differences are shown with a colored background. The nucleotide position numbers of the *COI* gene are shown above the alignment. See Tables S1 and S2 for detailed information on the haplotypes and samples included.

The combined dataset of haplotypes from this study and previous studies throughout the world consisted of 111 bisexual and 62 parthenogenetic haplotypes after trimming (Tables S1 and S2). Phylogenetic analysis revealed two distinct clades representing the two reproductive haplogroups (Figure 3; Figure S4). In each reproductive haplogroup, the selected candidate nucleotide position 1270 of the *COI* gene was consistent across all haplotypes (bisexual haplotypes with C, parthenogenetic haplotypes with T). Position 1294 was also consistently different between the two haplogroups. Meanwhile, although several other positions were mostly consistent, there were variations across haplotypes.

**Figure 4:**
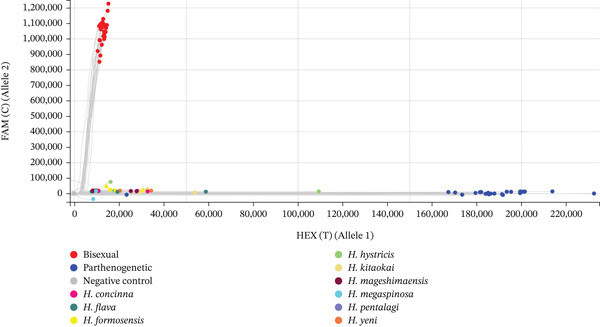
Allelic discrimination plots for *H. longicornis* and nine other *Haemaphysalis* species, using the newly designed real‐time PCR–based assay.

The melt curve analysis revealed a single peak at 76.8°C (±0.4°C), and no additional peaks were observed (Figure S5). The allelic discrimination plot showed two clearly separated clusters of bisexual and parthenogenetic *H. longicornis* (Figure 4). One individual assigned to the parthenogenetic clade by *COI* barcoding showed low fluorescence for both dyes. Note that the DNA concentration of this individual was low (< 0.025 ng/*μ*L). All other species showed little to no amplification for both probes. One *H. hystricis* individual showed intermediate HEX fluorescence but was considerably lower compared to all parthenogenetic *H. longicornis* individuals. Although different DNA extraction methods were used depending on specimen availability, the study was not designed to quantitatively compare extraction efficiency or real‐time PCR sensitivity between methods. Nevertheless, both methods produced successful amplification and genotyping results suitable for haplotype determination.

## 4. Discussion

### 4.1. National Distribution of the Two Reproductive Groups of *H. longicornis*

The national distribution of bisexual and parthenogenetic populations of *H. longicornis* showed mostly consistent results compared to previous investigations by Kitaoka [8] and Takada [17]. Specifically, parthenogenetic populations were distributed throughout Japan, while bisexual populations were distributed from central to southern Japan. Interestingly, Takada [17] showed the presence of males and females (i.e., a bisexual population) on Ishigaki Island of Okinawa Prefecture. However, only parthenogenetic individuals were confirmed in the present study. In 1933, three female *H. longicornis* (as *H. bispinosa*) were found on a cow on Okinawa Island [28], making this the first record of the species in Okinawa Prefecture. Similarly, one case of *H. longicornis* infestation on a cow was recorded in 1974 at Yonaguni Island, Okinawa Prefecture, located to the west of Ishigaki Island, although the stage and sex are unknown. These findings are suggested to be incidental findings where ticks were transported along with cattle [29]. Later in 1991, several *H. longicornis* were found on Yonaguni Island following the eradication of *Rhipicephalus microplus*. Due to the absence of males and the ability of reared females to reproduce without contact with males, these ticks were confirmed to be parthenogenetic populations [29]. Combined with results from this study, it is suggested that only parthenogenetic populations are distributed in Okinawa Prefecture. Takada′s finding may have been incidental, similar to previous findings of the tick on imported cattle. Another possibility is that parthenogenetic males, which are found in an extremely low ratio (1 male:400–1500 females), were confirmed [3, 30]. However, there is no detailed information regarding the finding of male *H. longicornis* in the region, and further discussions are not possible.

Parthenogenetic individuals were found throughout the Kyushu area, with some locations consisting of completely parthenogenetic populations. In contrast, Kitaoka [8] found both males and females in pastures throughout the Kyushu area. Meanwhile, an investigation of ticks on cattle throughout the Kyushu area carried out in the 1950s–1970s found *H. longicornis* at 42 farms, of which only 19 included males [31]. At that time, the authors suspected the presence of parthenogenetic populations. Notably, most male *H. longicornis* were found at farms in Nagasaki and Kumamoto prefectures, while males were only confirmed at two of 19 farms in Miyazaki and Kagoshima prefectures. Results from this study are similar to those of Furuya and Iwashina [31], although both reproductive groups were found in each prefecture. It is, therefore, suggested that parthenogenetic populations are dominant in the southern parts of the Kyushu area, while bisexual populations are dominant in the central to western parts. It is unclear why Kitaoka found males throughout pastures of Kyushu while Furuya and Iwashina found few farms with males, despite investigations being carried out during roughly the same period and in similar habitats (cattle pastures and cattle). One possibility is that *Haemaphysalis yeni* may have been misidentified as *H. longicornis* at the time. *Haemaphysalis yeni* was first found in Yakushima, Kagoshima Prefecture in 1971 [32], only a few years before Kitaoka published his study. In a more recent study, *H. yeni* was found in three distinct areas of Miyazaki Prefecture [33]. Adult *H. yeni* and *H. longicornis* can be morphologically distinguished relatively easily [32, 34], but the former may have been overlooked since it was not yet known to be distributed in mainland Kyushu. In any case, few individuals have been molecularly investigated in many parts of Kyushu, and further investigations are needed to further discuss the distribution pattern in the Kyushu area.

Although some discrepancies were seen, the distribution pattern of each reproductive group revealed in this investigation did not largely differ from those of previous studies. Hence, while the distribution range and ratio of the two reproductive groups may possibly be shifting when observed at a finer scale, the overall distribution pattern in Japan has not seemed to have largely changed. This distribution pattern with bisexual populations being limited to central to southern Japan while parthenogenetic populations being distributed throughout Japan is suggested to have been shaped by several influxes of *H. longicornis* from continental Asia at different times [14]. Particularly, the ability of parthenogenetic populations to spread more rapidly is likely an important factor shaping the distribution of *H. longicornis* [1]. Additionally, Kitaoka [6] noted that parthenogenetic individuals were able to successfully develop at low temperatures, while bisexual individuals usually developed at 30°C–32°C, further suggesting that this difference in temperature resistance may have formed the apparent difference in distribution.

The coexistence of both bisexual and parthenogenetic populations was confirmed in many areas from central to western Japan. Such coexistence has been previously reported in parts of Japan and China [1, 14]. Interestingly, proportions of parthenogenetic individuals seemed to differ between locations, although these may potentially vary between years or seasons. Additionally, coexisting and non‐coexisting populations (i.e., only bisexual or parthenogenetic) were found in close proximity. This pattern was also observed in parts of China [1]. Such distribution patterns may be shaped by various factors such as climate, geographic barriers, land use, and host distribution [12, 35]. However, there is still insufficient data to discuss the underlying mechanisms of coexistence and factors related to small‐scale distribution patterns.

### 4.2. Validity of the Real‐Time PCR–Based Method to Distinguish the Two Reproductive Groups

The real‐time PCR assay developed in this study could accurately distinguish between the bisexual and parthenogenetic haplogroups among ticks collected throughout Japan. The single peak during the melt curve analysis revealed that the designed PCR primers (Hlongi_coi_F6 and Hlongi_coi_R6) specifically amplified the target region. The allelic discrimination plot showed clearly separated clusters resembling the two reproductive haplogroups, showing that the real‐time PCR assay can efficiently identify the reproductive haplogroups. Additionally, little to no amplification was seen for all other species. Some *Haemaphysalis* species such as *H. mageshimaensis* and *H. yeni*, both distributed in Japan, are morphologically very similar to *H. longicornis*, particularly during larval and nymphal stages [36, 37]. Results from this study suggest that the sensitivity of this real‐time assay is high enough for simultaneous screening of possibly misidentified tick species. However, one parthenogenetic *H. longicornis* exhibited low fluorescence for both dyes, potentially due to poor DNA quality or low concentration, as the DNA concentration of this sample was < 0.025 ng/*μ*L. Some optimizations such as increasing DNA input and increasing cycles could improve the output. Furthermore, qPCR results should always be backed up by morphological observations to confirm the tick species. Although the real‐time PCR assay developed in this study does not directly determine ploidy, previous studies have demonstrated a close correspondence between mitochondrial haplogroups and reproductive type using flow cytometry and SNP analyses [1, 9]. Therefore, this assay provides a rapid and practical method for identifying the mitochondrial haplogroups corresponding to the two reproductive groups.

## 5. Conclusion

This study updates the national distribution of the two reproductive forms of *H. longicornis* in Japan, using a molecular approach. Parthenogenetic individuals were confirmed throughout the country, while bisexual individuals remained limited to central and western regions, mostly consistent with earlier surveys based on sex bias per population. Coexisting and single‐form populations were found in close proximity, highlighting the need for fine‐scale monitoring. Continued investigations throughout Japan at a finer scale are essential to track the distribution of bisexual and parthenogenetic populations of *H. longicornis*. While previous studies have suggested that parthenogenetic populations may play an important role in the spread of tick‐borne pathogens, limited information is available regarding possible differences in the epidemiological roles of the two reproductive groups, and further investigations are needed. Furthermore, future studies incorporating additional molecular markers may further clarify the evolutionary relationships among *H. longicornis* populations, including the biological significance of the undefined haplotypes observed in this study.

The newly developed real‐time PCR–based assay will serve as a rapid tool to distinguish the two reproductive haplogroups of *H. longicornis*, which is important for further understanding of differences in population dynamics and evolutionary adaptability, as well as their role in disease transmission. The assay can also serve as a reliable method to check possible morphological misidentifications of morphologically similar *Haemaphysalis* species. Furthermore, this method could help uncover potential shifts in distributions of each reproductive group. Importantly, the diagnostic nucleotide position selected for the probes was consistent across all investigated haplotypes, including those that have only been found outside of Japan. Therefore, although the real‐time PCR assay could only be tested using samples from Japan, this method is suggested to be able to accurately distinguish the two haplogroups for *H. longicornis* of other countries as well. Additional data from other countries and regions would further strengthen the global applicability of this assay.

## Funding

This study was funded by the Japan Agency for Medical Research and Development, 10.13039/100009619, JP23fk0108613, JP23fk0108625, JP23wm0225030, JP24fk0108693, and JP25fk0108717, and the Japan Society for the Promotion of Science, 10.13039/501100001691, JP25KJ2130.

## Conflicts of Interest

The authors declare no conflicts of interest.

## Supporting Information

Additional supporting information can be found online in the Supporting Information section.

## Supporting information


**Supporting Information 1** Table S1: Detailed sample information of *Haemaphysalis longicornis* ticks investigated in this study. Note that accession numbers were only assigned to unique full‐length (659 bp) haplotypes per prefecture. Upon aligning and trimming, new names (“aligned haplotype names”) were assigned to each unique haplotype, used as reference for Figure S1. Table S2: Sample information of *Haemaphysalis longicornis* ticks from previous studies. Note that only one accession number is shown for each haplotype of each reference (i.e., accession numbers are not listed for identical haplotypes). Upon aligning and trimming, new names (“aligned haplotype names”) were assigned to each unique haplotype, used as references for Figure S1. Aligned haplotype names including “‐j” are those not found in individuals of this study but found in other samples from Japan. Aligned haplotype names including “‐f” are those found only outside of Japan. Table S3: Detailed sample information of nine *Haemaphysalis* tick species used to test the qPCR assay in this study.

**Supporting Information 2** Figure S1: Maximum likelihood tree of *COI* sequences (659 bp) of *Haemaphysalis longicornis* obtained in this study, constructed using the Tamura‐3‐parameter model. Bisexual and parthenogenetic groups are marked based on haplotypes from a previous study, which are shown in red (bisexual) and blue (parthenogenetic) [1]. *Haemaphysalis hystricis* was used as an outgroup. Haplotypes from this study are shown in bold. Haplotype names are followed by parentheses containing accession numbers and sample numbers. L = larva, N = nymph, F = female, M = male. Haplotypes from males are marked with a triangle (◀). The haplotypes that could not be defined to a specific haplogroup are shown in yellow.

**Supporting Information 3** Figure S2: Distribution map of *H. longicornis* in Japan. The top left and middle are recreated from figures in previous studies [8, 17]. The top right and all bottom maps are created based on data from the present study and from previous studies utilizing *COI* barcoding for identification of the reproductive groups [9, 14]. Note that in the recreated maps, points do not show sample sizes. The areas marked with dotted lines are shown in more detail in Figure S3.

**Supporting Information 4** Figure S3: Details of the distribution map for *H. longicornis* in Japan, based on data from the present study and from previous studies utilizing *COI* barcoding for identification of the reproductive groups [9, 14].

**Supporting Information 5** Figure S4: Maximum likelihood tree of *COI* sequences (602 bp) of *Haemaphysalis longicornis*, constructed using the Tamura‐3‐parameter model, and multiple alignment of the amplification region of the real‐time PCR assay developed in this study. Bisexual and parthenogenetic groups are marked based on haplotypes from a previous study [1]. Haplotypes in which the ploidy has been confirmed by flow cytometry or SNP analysis are labeled with an asterisk (∗). Haplotypes that could not be defined to a specific haplogroup are shown in light color. *Haemaphysalis hystricis* was used as an outgroup. In the multiple alignment, dots (“・”) indicate positions identical to the top haplotype (par‐j07), while nucleotide differences are shown with a colored background. Primer and probe regions are marked with a gray and black outline, respectively. See Tables S1 and S2 for detailed information on the haplotypes and samples included.

**Supporting Information 6** Figure S5: Derivative melt curve analysis for *Haemaphysalis longicornis*, using the designed primers Hlongi_coi_F6 and Hlongi_coi_R6.

## Data Availability

The data that support the findings of this study are available in the supporting information of this article.
