# Supplementary material for: National Distribution of Bisexual and Parthenogenetic Haemaphysalis longicornis of Japan, and a Real‐Time PCR–Based Method to Distinguish the Two Reproductive Groups
Source: J Parasitol Res. 2026 Jul 31;2026:9395344. doi: 10.1155/japr/9395344 (PMC13426480; doi:10.1155/japr/9395344)
Supplement: Supplementary file 2 — Supporting Information 2 Figure S1: Maximum likelihood tree of COI sequences (659 bp) of Haemaphysalis longicornis obtained in this study, constructed using the Tamura‐3‐parameter model. Bisexual and parthenogenetic groups are marked based on haplotypes from a previous study, which are shown in red (bisexual) and blue (parthenogenetic) [1]. Haemaphysalis hystricis was used as an outgroup. Haplotypes from this study are shown in bold. Haplotype names are followed by parentheses containing accession numbers and sample numbers. L = larva, N = nymph, F = female, M = male. Haplotypes from males are marked with a triangle (◀). The haplotypes that could not be defined to a specific haplogroup are shown in yellow. [file JAPR-2026-9395344-s002.pdf]

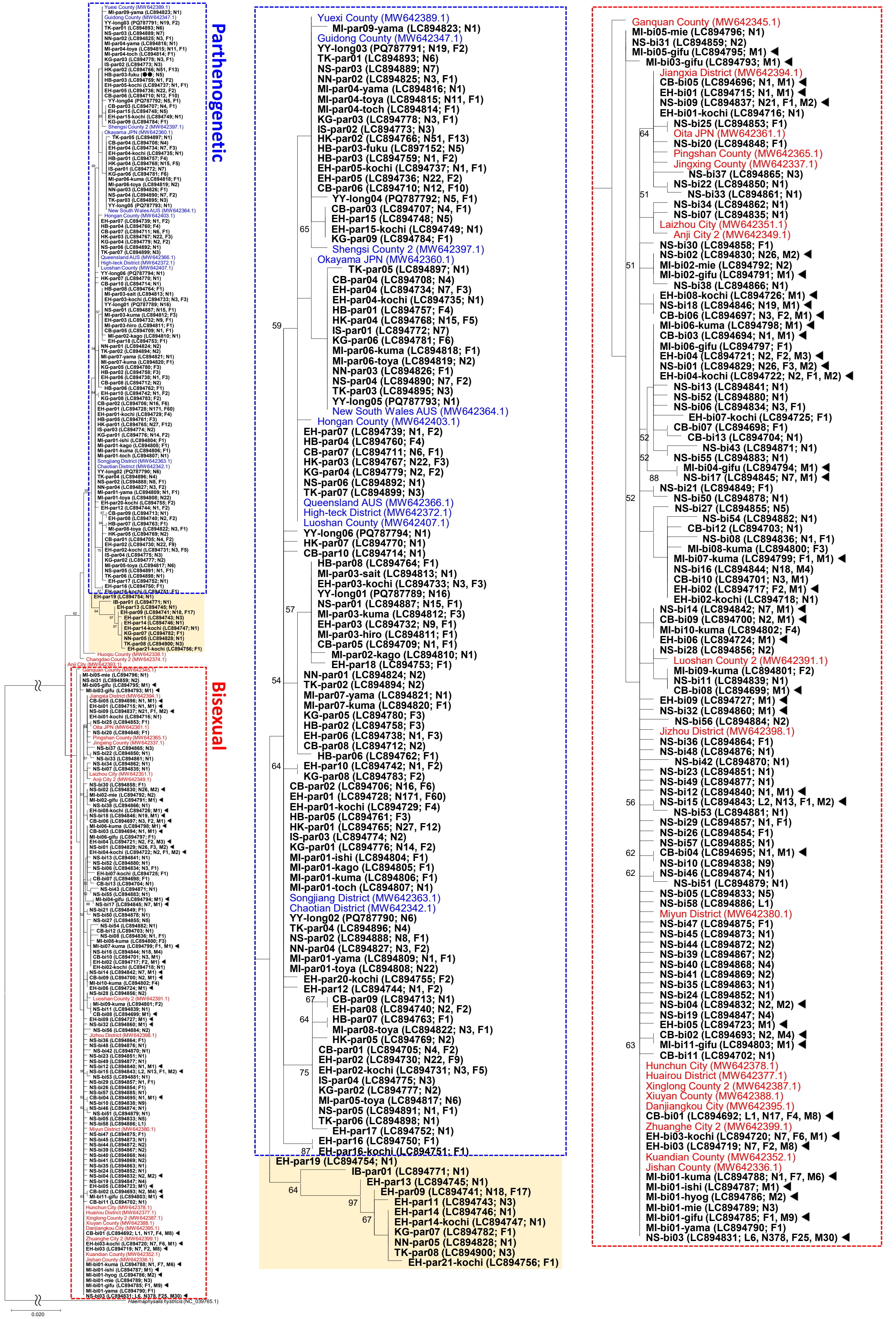

Parthenogenetic
Bisexual
Yuexi County (MW642389.1)
MI-par09-yama (LC894823; N1)
Guidong County (MW642347.1)
YY-long03 (PQ787791; N19, F2)
TK-par01 (LC894893; N6)
NS-par03 (LC894889; N7)
NN-par02 (LC894825; N3, F1)
MI-par04-yama (LC894816; N1)
MI-par04-toya (LC894815; N11, F1)
MI-par04-toch (LC894814; F1)
KG-par03 (LC894778; N3, F1)
IS-par02 (LC894773; N3)
HK-par02 (LC894766; N51, F13)
HB-par03-fuku (LC897152; N5)
HB-par03 (LC894759; N1, F2)
EH-par05-kochi (LC894737; N1, F1)
EH-par05 (LC894736; N22, F2)
CB-par06 (LC894710; N12, F10)
YY-long04 (PQ787792; N5, F1)
CB-par03 (LC894707; N4, F1)
EH-par15 (LC894748; N5)
EH-par15-kochi (LC894749; N1)
KG-par09 (LC894784; F1)
Shengsi County 2 (MW642397.1)
Okayama JPN (MW642360.1)
TK-par05 (LC894897; N1)
CB-par04 (LC894708; N4)
EH-par04 (LC894734; N7, F3)
EH-par04-kochi (LC894735; N1)
HB-par01 (LC894757; F4)
HK-par04 (LC894768; N15, F5)
IS-par01 (LC894772; N7)
KG-par06 (LC894781; F6)
MI-par06-kuma (LC894818; F1)
MI-par06-toya (LC894819; N2)
NN-par03 (LC894826; F1)
NS-par04 (LC894890; N7, F2)
TK-par03 (LC894895; N3)
YY-long05 (PQ787793; N1)
New South Wales AUS (MW642364.1)
Hongan County (MW642403.1)
EH-par07 (LC894739; N1, F2)
HB-par04 (LC894760; F4)
CB-par07 (LC894711; N6, F1)
HK-par03 (LC894767; N22, F3)
KG-par04 (LC894779; N2, F2)
NS-par06 (LC894892; N1)
TK-par07 (LC894899; N3)
Queensland AUS (MW642366.1)
High-teck District (MW642372.1)
Luoshan County (MW642407.1)
YY-long06 (PQ787794; N1)
HK-par07 (LC894770; N1)
CB-par10 (LC894714; N1)
HB-par08 (LC894764; F1)
MI-par03-sait (LC894813; N1)
EH-par03-kochi (LC894733; N3, F3)
YY-long01 (PQ787789; N16)
NS-par01 (LC894887; N15, F1)
MI-par03-kuma (LC894812; F3)
EH-par03 (LC894732; N9, F1)
MI-par03-hiro (LC894811; F1)
CB-par05 (LC894709; N1, F1)
MI-par02-kago (LC894810; N1)
EH-par18 (LC894753; F1)
NN-par01 (LC894824; N2)
TK-par02 (LC894894; N2)
MI-par07-yama (LC894821; N1)
MI-par07-kuma (LC894820; F1)
KG-par05 (LC894780; F3)
HB-par02 (LC894758; F3)
EH-par06 (LC894738; N1, F3)
CB-par08 (LC894712; N2)
HB-par06 (LC894762; F1)
EH-par10 (LC894742; N1, F2)
KG-par08 (LC894783; F2)
CB-par02 (LC894706; N16, F6)
EH-par01 (LC894728; N171, F60)
EH-par01-kochi (LC894729; F4)
HB-par05 (LC894761; F3)
HK-par01 (LC894765; N27, F12)
IS-par03 (LC894774; N2)
KG-par01 (LC894776; N14, F2)
MI-par01-ishi (LC894804; F1)
MI-par01-kago (LC894805; F1)
MI-par01-kuma (LC894806; F1)
MI-par01-toch (LC894807; N1)
Songjiang District (MW642363.1)
Chaotian District (MW642342.1)
YY-long02 (PQ787790; N6)
TK-par04 (LC894896; N4)
NS-par02 (LC894888; N8, F1)
NN-par04 (LC894827; N3, F2)
MI-par01-yama (LC894809; N1, F1)
MI-par01-toya (LC894808; N22)
EH-par20-kochi (LC894755; F2)
EH-par12 (LC894744; N1, F2)
CB-par09 (LC894713; N1)
EH-par08 (LC894740; N2, F2)
HB-par07 (LC894763; F1)
MI-par08-toya (LC894822; N3, F1)
HK-par05 (LC894769; N2)
CB-par01 (LC894705; N4, F2)
EH-par02 (LC894730; N22, F9)
EH-par02-kochi (LC894731; N3, F5)
IS-par04 (LC894775; N3)
KG-par02 (LC894777; N2)
MI-par05-toya (LC894817; N6)
NS-par05 (LC894891; N1, F1)
TK-par06 (LC894898; N1)
EH-par17 (LC894752; N1)
EH-par16 (LC894750; F1)
EH-par16-kochi (LC894751; F1)
EH-par19 (LC894754; N1)
IB-par01 (LC894771; N1)
EH-par13 (LC894745; N1)
EH-par09 (LC894741; N18, F17)
EH-par11 (LC894743; N3)
EH-par14 (LC894746; N1)
EH-par14-kochi (LC894747; N1)
KG-par07 (LC894782; F1)
NN-par05 (LC894828; N1)
TK-par08 (LC894900; N3)
EH-par21-kochi (LC894756; F1)
Huoqiu County (MW642338.1)
Changdao County 2 (MW642374.1)
Anji City (MW642393.1)
Ganquan County (MW642345.1)
MI-bi05-mie (LC894796; N1)
NS-bi31 (LC894859; N2)
MI-bi05-gifu (LC894795; M1)
MI-bi03-gifu (LC894793; M1)
Jiangxia District (MW642394.1)
CB-bi05 (LC894696; N1, M1)
EH-bi01 (LC894715; N1, M1)
NS-bi09 (LC894837; N21, F1, M2)
EH-bi01-kochi (LC894716; N1)
NS-bi25 (LC894853; F1)
Oita JPN (MW642361.1)
NS-bi20 (LC894848; F1)
Pingshan County (MW642365.1)
Jingxing County (MW642337.1)
NS-bi37 (LC894865; N3)
NS-bi22 (LC894850; N1)
NS-bi33 (LC894861; N1)
NS-bi34 (LC894862; N1)
NS-bi07 (LC894835; N1)
Laizhou City (MW642351.1)
Anji City 2 (MW642349.1)
NS-bi30 (LC894858; F1)
NS-bi02 (LC894830; N26, M2)
MI-bi02-mie (LC894792; N2)
MI-bi02-gifu (LC894791; M1)
NS-bi38 (LC894866; N1)
EH-bi08-kochi (LC894726; M1)
NS-bi18 (LC894846; N19, M1)
CB-bi06 (LC894697; N3, F2, M1)
MI-bi06-kuma (LC894798; M1)
CB-bi03 (LC894694; N1, M1)
MI-bi06-gifu (LC894797; F1)
EH-bi04 (LC894721; N2, F2, M3)
NS-bi01 (LC894829; N26, F3, M2)
EH-bi04-kochi (LC894722; N2, F1, M2)
NS-bi13 (LC894841; N1)
NS-bi52 (LC894880; N1)
NS-bi06 (LC894834; N3, F1)
EH-bi07-kochi (LC894725; F1)
CB-bi07 (LC894698; F1)
CB-bi13 (LC894704; N1)
NS-bi43 (LC894871; N1)
NS-bi55 (LC894883; N1)
MI-bi04-gifu (LC894794; M1)
NS-bi17 (LC894845; N7, M1)
NS-bi21 (LC894849; F1)
NS-bi50 (LC894878; N1)
NS-bi27 (LC894855; N5)
NS-bi54 (LC894882; N1)
CB-bi12 (LC894703; N1)
NS-bi08 (LC894836; N1, F1)
MI-bi08-kuma (LC894800; F3)
MI-bi07-kuma (LC894799; F1, M1)
NS-bi16 (LC894844; N18, M4)
CB-bi10 (LC894701; N3, M1)
EH-bi02 (LC894717; F2, M1)
EH-bi02-kochi (LC894718; N1)
NS-bi14 (LC894842; N7, M1)
CB-bi09 (LC894700; N2, M1)
MI-bi10-kuma (LC894802; F4)
EH-bi06 (LC894724; M1)
NS-bi28 (LC894856; N2)
Luoshan County 2 (MW642391.1)
MI-bi09-kuma (LC894801; F2)
NS-bi11 (LC894839; N1)
CB-bi08 (LC894699; M1)
EH-bi09 (LC894727; M1)
NS-bi32 (LC894860; M1)
NS-bi56 (LC894884; N2)
Jizhou District (MW642398.1)
NS-bi36 (LC894864; F1)
NS-bi48 (LC894876; N1)
NS-bi42 (LC894870; N1)
NS-bi23 (LC894851; N1)
NS-bi49 (LC894877; N1)
NS-bi12 (LC894840; N1, M1)
NS-bi15 (LC894843; L2, N13, F1, M2)
NS-bi53 (LC894881; N1)
NS-bi29 (LC894857; N1, F1)
NS-bi26 (LC894854; F1)
NS-bi57 (LC894885; N1)
CB-bi04 (LC894695; N1, M1)
NS-bi10 (LC894838; N9)
NS-bi46 (LC894874; N1)
NS-bi51 (LC894879; N1)
NS-bi05 (LC894833; N5)
NS-bi58 (LC894886; L1)
Miyun District (MW642380.1)
NS-bi47 (LC894875; F1)
NS-bi45 (LC894873; N1)
NS-bi44 (LC894872; N2)
NS-bi39 (LC894867; N2)
NS-bi40 (LC894868; N4)
NS-bi41 (LC894869; N2)
NS-bi35 (LC894863; N1)
NS-bi24 (LC894852; N1)
NS-bi04 (LC894832; N2, M2)
NS-bi19 (LC894847; N4)
EH-bi05 (LC894723; M1)
CB-bi02 (LC894693; N2, M4)
MI-bi11-gifu (LC894803; M1)
CB-bi11 (LC894702; N1)
Hunchun City (MW642378.1)
Huairou District (MW642377.1)
Xinglong County 2 (MW642387.1)
Xiuyan County (MW642388.1)
Danjiangkou City (MW642395.1)
CB-bi01 (LC894692; L1, N17, F4, M8)
Zhuanghe City 2 (MW642399.1)
EH-bi03-kochi (LC894720; N7, F6, M1)
EH-bi03 (LC894719; N7, F2, M8)
Kuandian County (MW642352.1)
Jishan County (MW642336.1)
MI-bi01-kuma (LC894788; N1, F7, M6)
MI-bi01-ishi (LC894787; M1)
MI-bi01-hyog (LC894786; M2)
MI-bi01-mie (LC894789; N3)
MI-bi01-gifu (LC894785; F1, M9)
MI-bi01-yama (LC894790; F1)
NS-bi03 (LC894831; L6, N378, F25, M30)
Haemaphysalis hystricis (NC_039765.1)
0.020
