# Supplementary material for: National Distribution of Bisexual and Parthenogenetic Haemaphysalis longicornis of Japan, and a Real‐Time PCR–Based Method to Distinguish the Two Reproductive Groups
Source: J Parasitol Res. 2026 Jul 31;2026:9395344. doi: 10.1155/japr/9395344 (PMC13426480; doi:10.1155/japr/9395344)
Supplement: Supplementary file 3 — Supporting Information 3 Figure S2: Distribution map of H. longicornis in Japan. The top left and middle are recreated from figures in previous studies [8, 17]. The top right and all bottom maps are created based on data from the present study and from previous studies utilizing COI barcoding for identification of the reproductive groups [9, 14]. Note that in the recreated maps, points do not show sample sizes. The areas marked with dotted lines are shown in more detail in Figure S3. [file JAPR-2026-9395344-s003.pdf]

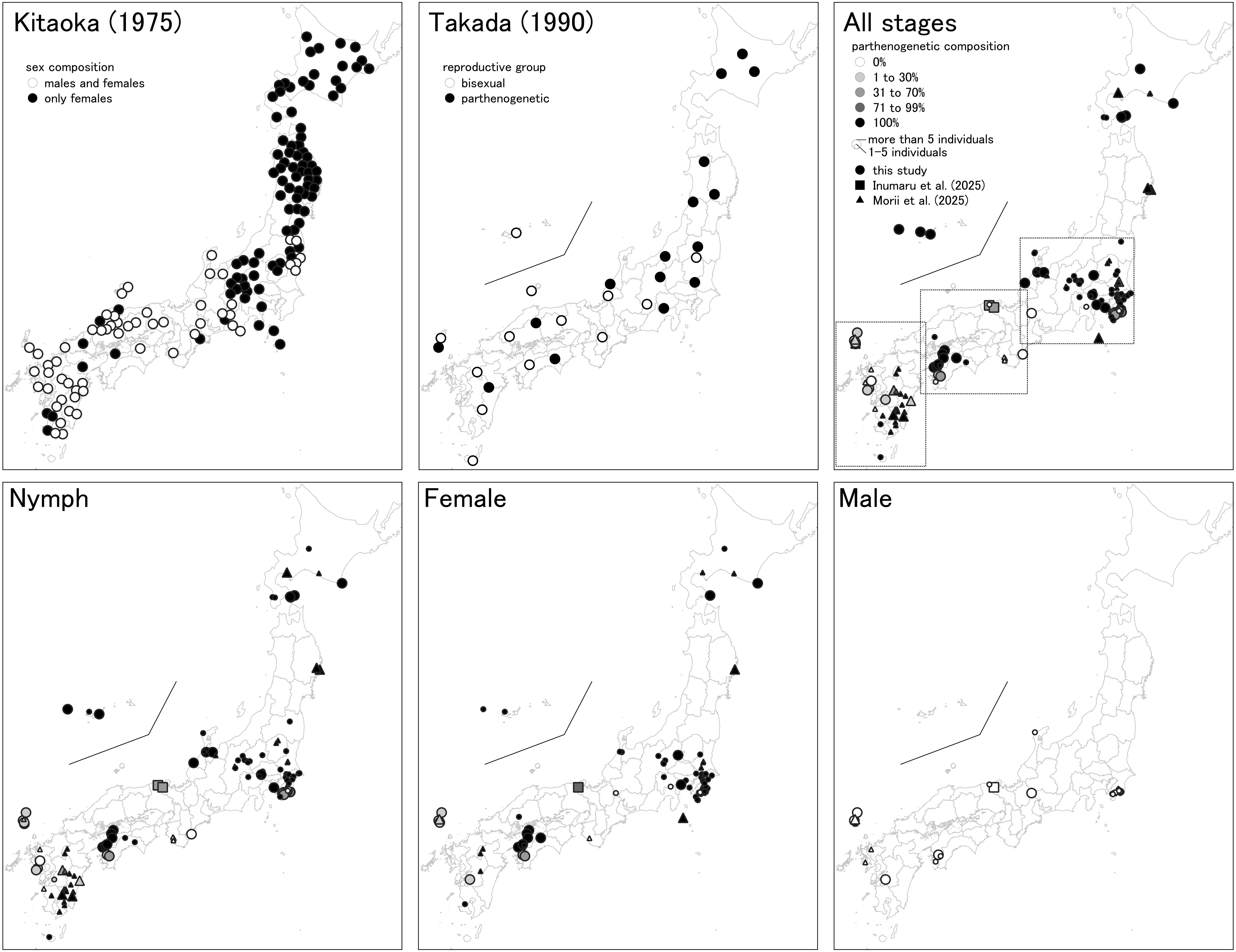
Kitaoka (1975)
sex composition
males and females
only females
Takada (1990)
reproductive group
bisexual
parthenogenetic
All stages
parthenogenetic composition
0%
1 to 30%
31 to 70%
71 to 99%
100%
more than 5 individuals
1–5 individuals
this study
Inumaru et al. (2025)
Morii et al. (2025)
Nymph
Female
Male
