# Supplementary material for: National Distribution of Bisexual and Parthenogenetic Haemaphysalis longicornis of Japan, and a Real‐Time PCR–Based Method to Distinguish the Two Reproductive Groups
Source: J Parasitol Res. 2026 Jul 31;2026:9395344. doi: 10.1155/japr/9395344 (PMC13426480; doi:10.1155/japr/9395344)
Supplement: Supplementary file 4 — Supporting Information 4 Figure S3: Details of the distribution map for H. longicornis in Japan, based on data from the present study and from previous studies utilizing COI barcoding for identification of the reproductive groups [9, 14]. [file JAPR-2026-9395344-s004.pdf]

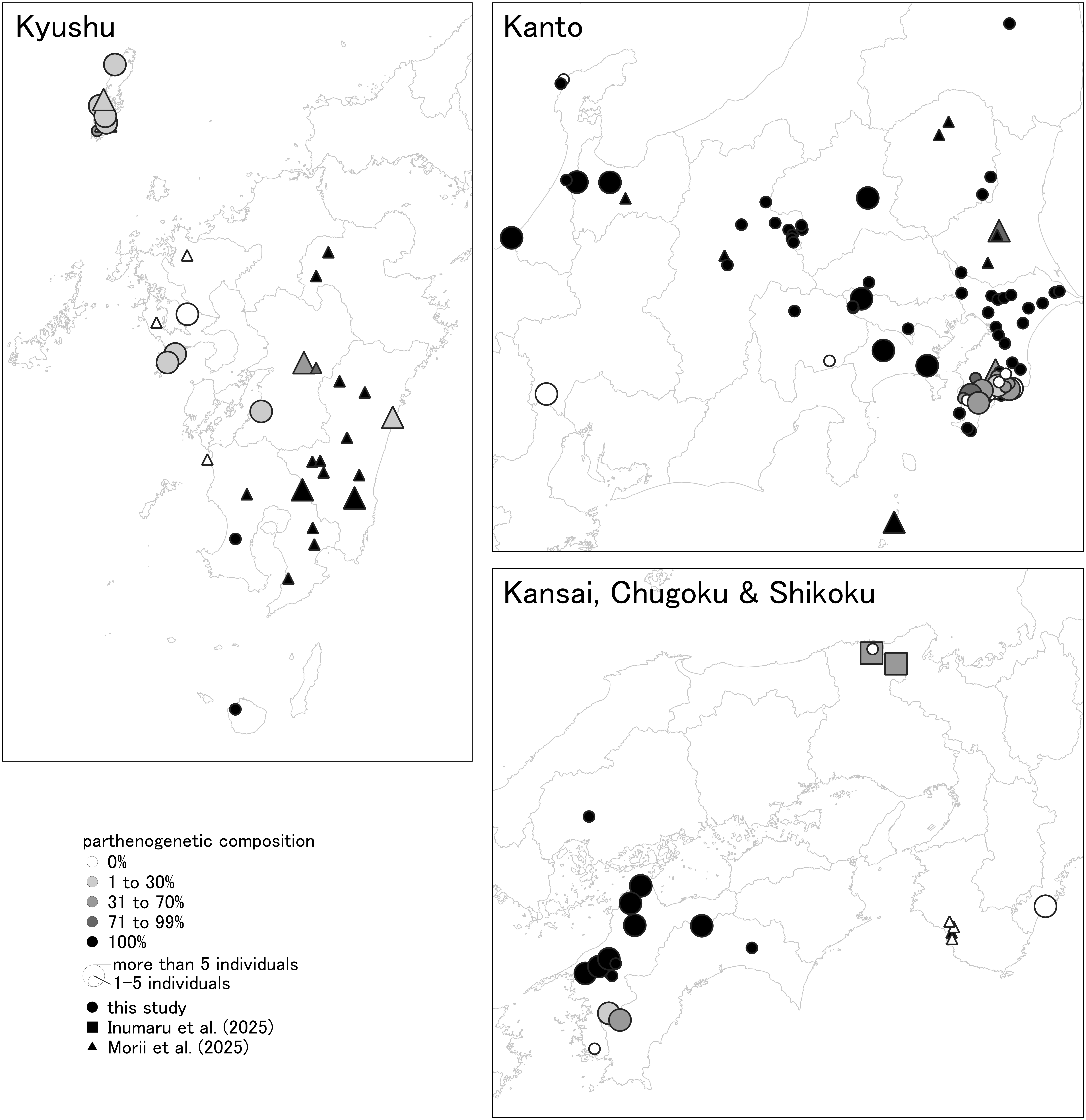
Kyushu
Kanto
Kansai, Chugoku & Shikoku
parthenogenetic composition
0%
1 to 30%
31 to 70%
71 to 99%
100%
more than 5 individuals
1–5 individuals
this study
Inumaru et al. (2025)
Morii et al. (2025)
