# Supplementary figures and images for: National Distribution of Bisexual and Parthenogenetic Haemaphysalis longicornis of Japan, and a Real‐Time PCR–Based Method to Distinguish the Two Reproductive Groups
Source: J Parasitol Res. 2026 Jul 31;2026:9395344. doi: 10.1155/japr/9395344 (PMC13426480; doi:10.1155/japr/9395344)

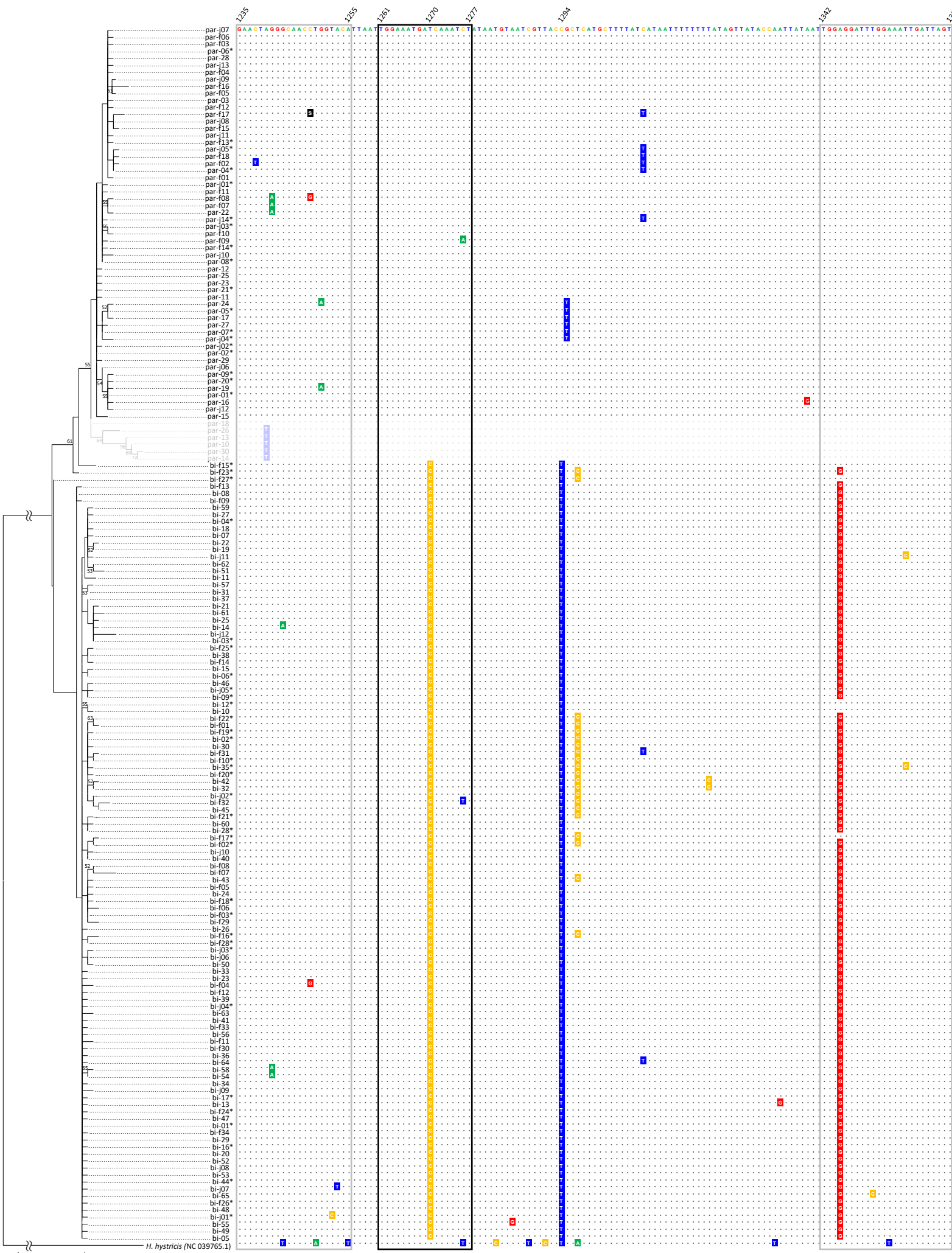

Supplement: Supplementary file 5 — Supporting Information 5 Figure S4: Maximum likelihood tree of COI sequences (602 bp) of Haemaphysalis longicornis, constructed using the Tamura‐3‐parameter model, and multiple alignment of the amplification region of the real‐time PCR assay developed in this study. Bisexual and parthenogenetic groups are marked based on haplotypes from a previous study [1]. Haplotypes in which the ploidy has been confirmed by flow cytometry or SNP analysis are labeled with an asterisk (∗). Haplotypes that could not be defined to a specific haplogroup are shown in light color. Haemaphysalis hystricis was used as an outgroup. In the multiple alignment, dots (“・”) indicate positions identical to the top haplotype (par‐j07), while nucleotide differences are shown with a colored background. Primer and probe regions are marked with a gray and black outline, respectively. See Tables S1 and S2 for detailed information on the haplotypes and samples included. [file JAPR-2026-9395344-s005.pdf]

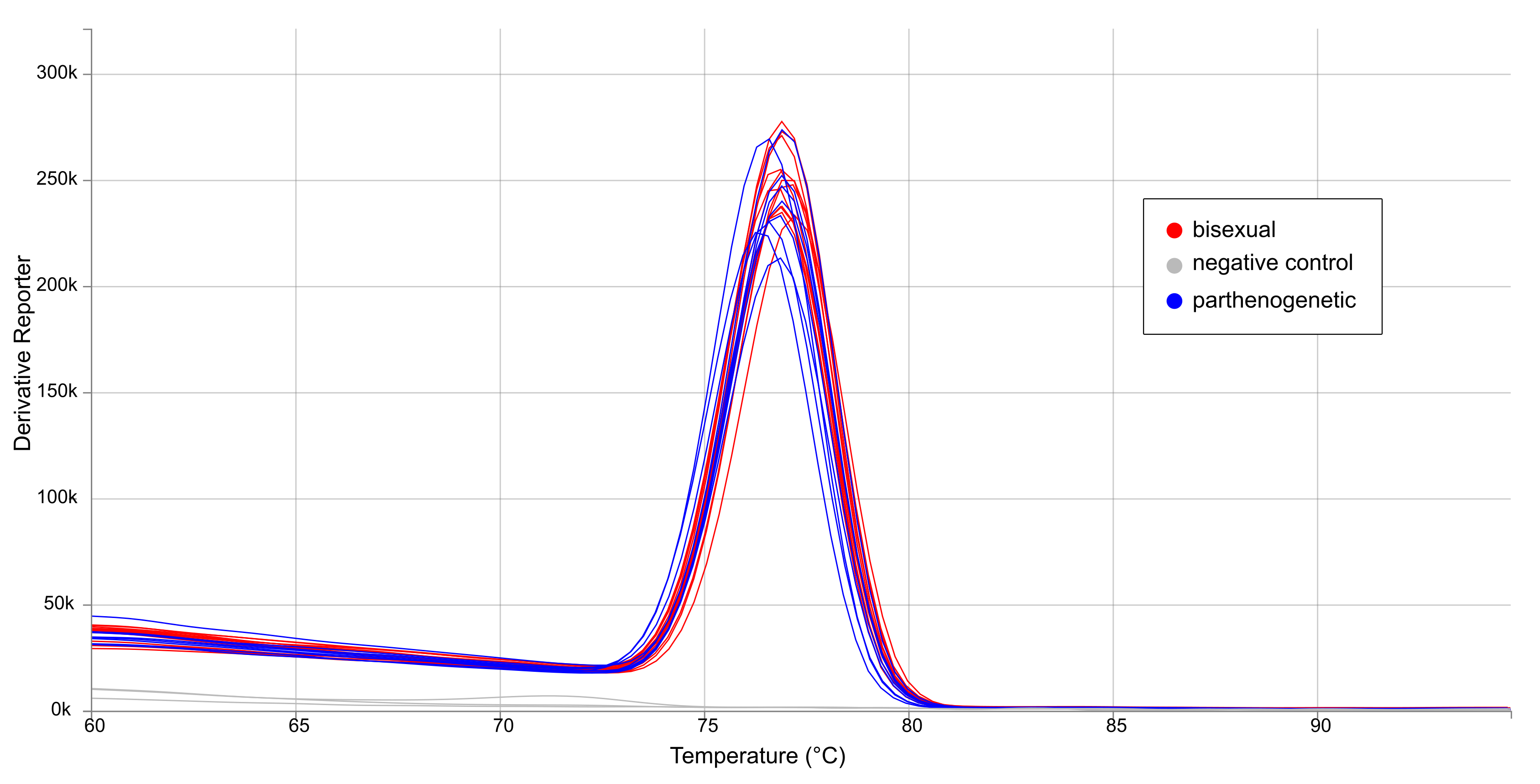

Supplement: Supplementary file 6 — Supporting Information 6 Figure S5: Derivative melt curve analysis for Haemaphysalis longicornis, using the designed primers Hlongi_coi_F6 and Hlongi_coi_R6. [file JAPR-2026-9395344-s006.png]
